# Teachers’ Views on the Participation of Parents in the Transition of their Children from Kindergarten to Primary School

**DOI:** 10.3390/bs9120124

**Published:** 2019-11-22

**Authors:** Marina Besi, Maria Sakellariou

**Affiliations:** Department of Early School Education, University of Ioannina, University Campus, P.O. box 1186, PC 45110 Ioannina, Greece; marisak@uoi.com

**Keywords:** kindergartens, teachers, parents, transition practices, Greece

## Abstract

Internationally, a great number of researchers have pointed out the significance of school–family relationship in the process of children’s transition to primary schools. However, only recently has it been a subject of research in Greece. The purpose of this particular research, which has a sample of 1602 pre-primary and primary school teachers, is to investigate teachers’ viewpoints on the role of parents in the process of their children’s transition to primary school. Data has been collected through the use of questionnaires. Statistical analysis has shown that the overwhelming majority of teachers consider cooperation with parents as necessary. They indicate that the most important factors for successful transition are parents’ level of trust in teachers, their views on schools and learning, and their support for the new situation their child experiences. As far as the most appropriate practices are concerned, almost all teachers mention updating parents at the beginning of the school year, while many suggest that parents and children visit primary schools and that common meetings of both kindergarten and primary school teachers with parents take place before school begins. It therefore seems that teachers acknowledge the role of parents in the process of transition and suggest appropriate practices for their participation.

## 1. Introduction

The transition of children from kindergarten to primary school is considered as one of the most critical periods of childhood. This is a very complex process of change and does not simply refer to the fact that the child goes from one building (kindergarten) to another (primary school). It requires adaptation to a new group, a new role, a new teacher, and new expectations [1]. 

The first day of elementary school is an exciting time, full of joy, excitement, and optimism, as well as anxiety and worry. Requirements change, and this can make children feel unsafe, embarrassed, and less protected [2]. Adaptation and confrontation to changes are important, as previous research efforts have shown that a positive beginning in school determines a child’s future educational experiences to some extent [3,4,5,6]. In recent years, the transition from the kindergarten to primary school has attracted considerable attention as a research topic in Greece [7,8,9,10]. 

The family–school relationship is critical at any stage of child’s education, but it becomes more important during the transition to primary school. Children’s attendance at the new and unfamiliar environment of primary school is a defining experience for their lives. During this process, children experience a multitude of changes, which extend over time after the transition. At the same time, parents are now becoming more active. They make every effort to prepare their children for the changes that are coming [2]. However, parents themselves have different expectations and requirements from school [11]. In the ecosystemic-developmental model, adaptation to a system outside the family environment is characterized by significant changes in identity, roles, and relationships. 

Parents can help children develop resilience to deal with the changes they will encounter [12]. Their role is very important in social, emotional and academic support they offer to their children at home and at school [13,14]. Parents’ active participation in the education of their children improves children’s attitude toward school while increasing motivation for learning [15].

The resilience framework is based on the principle that teachers and parents can be the most powerful external protective factors for children to deal with school-based transitions in their lifetime [16]. Mental resilience is not taught, but it can be facilitated and enhanced by positive educational experiences. This can be cultivated through the promotion of a sense of well-being, support of self-esteem, self-efficacy, and optimism. Teachers can teach children to recognize problems and solve them intelligently so that they can feel strong and able to confidently cope with new experiences [2,17]. The goal is for children to see themselves as strong and successful learners, to work together to solve problems, and deal with their environment in a changing world.

The role of teachers is crucial in reducing or eliminating the stress of adapting to the transition process by supporting parents in coping with stress and expectations through regular communication and update [2]

All systems—that is, the family, the school, and the community—complement, assist, and co-ordinate the transition process [2,14]. The ultimate goal of both family and school is to develop children’s self-esteem through self-improvement and to allow them to acquire the mental resilience, skills, and abilities necessary to support them throughout their lives [14].

Best practices in transition have been studied throughout the world and include family involvement in the transition. Parent supervision of their children’s day-to-day activities, assistance with their children’s homework, and the practices parents use to help their children develop motivation for learning and a positive attitude toward school are dimensions of parental involvement at home [18,19]. On the other hand, parent–teacher communication, parental involvement in school events, and/or in school management through a Parents and Guardians Association are elements of parental involvement in school [20]. However, the type of parental involvement that seems to have the most impact on students’ school performance is related to the parents’ physical presence at school and their involvement in school activities [21,22]. 

The foundation for good parent and teacher relationships is frequent and open communication, mutual respect, and a clear understanding of what is best for each child [23]. Communication is enhanced when parents and teachers are aware of their values, opinions, and expectations; their perceptions about children; and the values they attribute to education [24,25].

Schools and teachers need to recognize the variety of needs, expectations and diversity of families. They need to understand how parents think by listening to them, which allows them to develop supportive teaching and learning approaches. They also need to utilize “knowledge chapters” to enrich the school process and to validate parents in teacher roles in useful ways [26,27].

Cooperation between family and school is considered as an essential prerequisite so that the positive results of transition can be supported. In many studies, it has been shown that family–school cooperation enhances the educational experience of children [15,28,29] and promotes the development of their social skills [25,30,31]. 

When parents and schools work together and share a common understanding of transition, they can jointly plan an effective and positive experience for children who start attending primary school [21,28,32]. Parents and teachers, who create academic and social goals together, enhance continuity between home and school and facilitate the child’s transition from home to school [33]

The aim of this paper is to investigate the views of Greek teachers, especially their views of kindergarten and primary school teachers on the role of parents in the process of their children’s transition to primary school.

## 2. Materials and Methods

This paper is a part of a national research project and was achieved using a questionnaire. Quantitative research was chosen as it allows the collection of large volumes of data from a large sample of respondents and the connection of two or more characteristics [34]. In particular, a questionnaire of closed-ended questions was developed based on the research objective. The questions were written after studying the international literature. The questionnaire was structured in five sections. The first concerned the recording of the individual data of the respondents, in particular gender, years of teaching, educational level, position in the education system, and the educational district which they work in. The second section included questions about teachers’ views on transition from kindergartens to primary schools. The third section consisted of four sets of questions (50 sentences) and referred to the competences or skills that must be acquired by kindergarten students for a successful transition to primary school. The fourth section consisted of five questions referring to the factors related to successful transition of children from kindergarten to elementary school, and the fifth section lists appropriate practices for a successful transition of children and settles the differences between kindergartens and elementary schools.

The following different types of questions were used in the preparation of the questionnaire.

- Ranking questions: The respondent is asked to select the answers in order of priority, depending on which answer he or she deems most important, in order to record the experiences and preferences of the participants.
- Multiple Choice Questions: The respondent can select more than one answer that he or she agrees with.
- Scale Questions: Respondent is asked to rate a specific question category (five-point Likert scale where 1 = Strongly disagree, 2 = Disagree, 3 = Neither agree nor disagree, 4 = Agree, and 5 = Agree completely) in order to emphasize the intensity of the choices and to compare them.

The technique that was applied is the Proportional Stratified Sample Surveys [35]. According to this technique, the sample was divided according to the characteristics of the population in the layers (educational regions of Greece), and then random samples were selected from each layer. The stratified sample survey was designed to ensure the representation of all sections of the population, to reduce the estimation error and to have a sufficient number of subpopulation subjects. This technique generally leads to estimations with a high level of precision.

The questionnaires were distributed at training sessions. In the majority of cases, the researcher was present during the completion of the questionnaire, ensuring that all the questions were answered correctly, thus avoiding possible misinterpretations and data loss at the time of completion. In some cases, the researcher’s associates were present at the time of its completion. The sampling was based solely on the participants’ consent to respond to the questionnaire on the basis of their interest in the subject being negotiated. This ensured that all queries were answered and all questionnaires were returned.

The target population is teachers of primary education (kindergarten teachers, teachers of primary schools and directors of primary schools) of the 13 educational districts of Greece. The sample of the survey was selected by the laws of sampling and represents 4% of all kindergarten teachers, 4% of teachers of first and second class of the total of each educational district, and 4% of the directors of primary schools of the total of each educational district. The figures for the total number of teachers in each district were provided by the Ministry of Education. The dispersion of population observations played a decisive role in determining the size. This percentage was considered satisfactory by the researcher as being representative of the educational population of Greece. The final sample size was 1602 teachers—784 kindergarten teachers, 634 teachers, and 184 directors of primary schools. 

The data analysis provided by the primary data survey was done using the statistical analysis program SPSS 23.0 [36]. 

## 3. Results

A large percentage of the sample teachers (79.3%) point out that it is necessary to cooperate with the pupils’ parents and guardians in the process of transition.

Referring to the question of what requirements they feel should apply to parents in order to ensure a successful transition to primary school, almost three out of four teachers (74%) say that there is a need for mutual trust and respect among all the interested parties (teachers, children, and parents). Then, they point out the need for parents to participate in the transition process (41.8%) and suggest that children’s and family’s expectations for school (30%) should be taken into account (Table 1).

As for the factors that influence the successful outcome of children’s transition from kindergarten to primary school, according to the findings shown in Table 2, more than half of the teachers refer to the level of trust of parents to teachers (57.5%) and to parents’ opinion of school and learning (49.6%). Then, teachers agree that the educational level (42.3%), the socio-economic level (32.4%), and the culture and mother tongue of the parents (31.3%) influence the successful outcome of the transition of their children from kindergarten to the primary school.

The relationship between teachers’ perceptions of the prerequisites for a successful transition according to their place of employment was then explored. According to the results of the chi-square test, there is a statistically significant relationship between teachers’ responses and their position on whether a successful transition requires “mutual trust and respect among all involved parties (teachers, children, and parents)” (χ^2^ (df = 2, N = 1602) = 15.25, p < 0.001). Specifically, the percentage of principals (82.5%) is higher than that of kindergarten (76.5%) and primary school (74.1%) teachers.

There is also a statistically significant relationship between teachers’ responses that a successful transition requires “taking into account children’s and families’ expectations for school” and their place of employment (χ^2^ (df = 2, N = 1602) = 19, 42, p < 0.001). Specifically, it was found that principals (39.7%) agree more with this view, compared to kindergarten (32.3%) and primary school teachers (24.4%).

Concerning “parents ‘involvement in the transition process,” the results of the chi-square test showed that there is no statistically significant relationship between teachers’ employment and job status (χ^2^ (df = 2, N = 1602) = 1.38, p = 0.501).

Teachers were also asked how parents can prepare their children for primary school attendance. As can be seen from the findings in Table 3, more than four out of five teachers agree that parental support for the new situation experienced by their child facilitates their transition (82.1%), while more than half of the teachers (54%) agree that parents should read books to their children and play math games at home regularly in order to prepare them properly for their transition to Primary School.

The results of the chi-square test revealed a statistically significant relationship between teachers’ responses to whether “parents’ view of school and learning” influence children’s successful transition and their place of employment (χ^2^ (df = 2, N = 1600) = 8.61, p = 0.017). Specifically, there is a higher percentage of kindergarten teachers (53.32%) who believe that parents’ view of school and learning influences the successful outcome of the transition from kindergarten to primary school compared to teachers (45.9%) and primary school principals (47%).

There is also a statistically significant relationship between teachers’ responses and their job status (χ^2^ (df = 2, N = 1602) = 7.77, p = 0.021), with 38.8% of kindergarten teachers considering that the educational level of parents influences children’s successful transition, compared to 45.3% of the primary school teachers and 46.7% of the primary school principals.

Regarding the “socio-economic level of the family,” there is a statistically significant relationship between teachers’ responses and their place of employment (χ^2^ (df = 2, N = 1602) = 10.62, p = 0.005), namely, the smaller percentage of kindergarten teachers (28.6%), compared to primary school teachers (36.6%) and primary school principals (34.2%).

On the other hand, there is no statistically significant relationship between teachers’ responses to whether “parents’ level of confidence in teachers” (χ^2^ (df = 2, N = 1601) = 3.90, p = 0.142) and “culture and language (ethnic minorities)” (x^2^ (df = 2, N = 1602) = 2.87, p = 0.238) influence the successful outcome of the transition and their place of employment.

Accordingly, fewer than half of the teachers (48.2%) agree that active participation of parents in the school program, dedicating time at home to help their children do their homework (40.1%), and help them learn to read (37.5%) are necessary in a transition program from kindergarten to primary school.

According to the chi-square test, there is a statistically significant relationship between teachers’ responses that “parents’ support for their child’s new situation facilitates their transition from kindergarten to primary school” and their job position (χ^2^ (df = 2, N = 1601) = 28.87, p < 0.001). Specifically, it was found that kindergarten teachers (87.4%) are more in line with this view than teachers (77.6%) and primary school principals (75.5%).

There is also a statistically significant relationship between teachers’ responses and their job status (χ^2^ (df = 2, N = 1602) = 9.44, p = 0.009), with the proportion of primary school principals (43.5%) being lower than that of kindergarten teachers (54.8%) and primary school teachers (56%).

Furthermore, the percentage of teachers (38.8%) who consider that active parental involvement in the school program is necessary in a transition program from kindergarten to primary school is lower than that of teachers (55.1%) and primary school principals (51.1%) (χ^2^ (df = 2, N = 1602) = 38.01, p < 0.001).

The chi-square test revealed a statistically significant relationship between teachers’ responses that “parents should provide time each day to assist their children in school activities at home” and their place of employment [x^2^ (df = 2, N = 1602) = 18.26, p < 0.001]. There is a higher proportion of teachers (46.5%) who have this view than kindergarten teachers (35.5%) and primary school principals (38%).

Similarly, the percentage of kindergarten teachers (30.6%) who believe that parents should learn how to help their children learn to read is better than primary school teachers (45.1%) and principals (40%) [χ^2^ (df = 2, N = 1602) = 32.37, p < 0.001].

Finally, teachers answered on which the best practices for parents are in order to facilitate the transition of their children from Kindergarten to Primary School. Their answers were quite varied, as it is shown in Table 4.

The most appropriate practice is considered when parents are invited to primary schools for briefing at the beginning of the school year. In fact, more than nine out of ten teachers report that they agree (42.2%) or totally agree (48.7%) that the practice “Parents are invited to primary schools for briefing at the beginning of the school year” is appropriate for the transition of children to Primary schools.

As far as the practices “Parents and children visit primary school before the beginning of the school year”, “Joint meetings of kindergarten and primary school teachers with parents before starting Primary school” and “Meetings and communication between the parents of the children of Kindergarten and Primary School” are concerned, teachers report relatively moderate to high levels of agreement regarding their appropriateness.

A two-way analysis of variance was performed with an “independent” variable of service position and a “dependent” variable of each of the above practices.

It was found that the main effect of the job position was statistically significant with principals agreeing more with the appropriateness of the practice “parents are invited to primary school for updating at the beginning of the year” compared to teachers (p = 0.047) (F (2, N = 1602) = 4.33, p = 0.013).

The main effect of job position was found to be statistically significant in the practice of “parents and children visiting primary school before the start of the school year” (F (2, N = 1602) = 63.16, p < 0.001). Kindergarten teachers agree more with the appropriateness of this practice, compared to primary school teachers (p < 0.001) and primary school principals (p = 0.002). Furthermore, primary school teachers were found to be less in agreement with primary school principals (p < 0.001).

The main effect of job position was found to be statistically significant for the practice of “common meetings of kindergarten and teacher with parents before school starts” (F (2, N = 1602) = 11.89, p < 0.001). Specifically, teachers agreed less with the appropriateness of this practice compared to kindergarten teachers (p < 0.001) and primary school principals (p < 0.001).

In addition, kindergarteners are more in agreement with the appropriateness of the practice of “meetings and communication between parents of kindergarten and primary school children,” compared to primary school teachers (p < 0.001). This relationship is statistically significant (F (2, N = 1602) = 9.71, p < 0.001).

The main effect of job position was also found to be statistically significant for the appropriateness of the “brochures or letters to family” practice (F (2, N = 1602) = 5.92, p = 0.003). Specifically, kindergarten teachers are more in line with this practice than primary school teachers.

On the contrary, it was found that the main effect of service position (F (2, N = 1600) = 1.30, p = 0.273) was not statistically significant in relation to the appropriateness of “telephone communication with family.”

## 4. Discussion and Conclusions

The results of this research are in line with previous research efforts [5,37] that have shown that co-operation between kindergarten and primary school teachers and the simultaneous familiarization of family can lead to a successful transition. Many states have made efforts and have seriously invested in developing transition programs, offering opportunities to establish strong relationships between children, their families, and schools. There are still many steps to be taken in Greece. However, it is recognized in the curriculum that parent–teacher co-operation relationships support the effective transition of children to primary school.

Sample teachers focus on mutual trust and respect between teachers and parents. Better and methodical home and school communication is likely to promote understanding on both sides and strengthen the relations of respect and trust, which are also underlined by research abroad [37,38,39]. It is very positive that teachers recognize the need to build relationships with their students’ parents. This may indicate suspicion and distrust of the work or boundaries of each side. A further qualitative survey could provide more complete information. The smooth transition from one educational level to another is a function of the quality of communication and cooperation between parents and teachers.

Particular emphasis is placed on the need for parents to participate in the transition process. That is, they adopt the ecosystemic model, which recognizes that the social contexts which children live in affect their development. School and family complement each other. The active involvement and interactions of individuals and systems shape the transition process, with the aim of ensuring the smooth entry and adaptation of infants to elementary school, ensuring continuity through activities that bridge the gap between family, kindergarten, and elementary school and linking child development with family. Supporting children’s transition involves mutual exchange of information, allowing teachers to acquire knowledge from families and children that will help them support pupils individually. By overcoming any obstacles (time, work, language, etc.), parents support their children considerably when they are actively involved in the transition process. 

The sample of the study agrees with the views of their colleagues abroad about the role of different expectations of parents and teachers in the transition process. Communicating their differences in their goals and expectations for school and children will contribute to the success of children in primary school [2,12,24,27]. Parents experience a change in their roles and expectations when their children move from kindergarten to elementary school. They have high expectations of their children attending school as they expect good academic performance, proper education and compliance with social behavior rules. They have the anxiety of adjustment and express their own expectations and concerns. They also want to be able to share and communicate their children’s needs, desires and thoughts. It is, of course, desirable and imperative to make clear what the expectations of parents for kindergarten teachers and of kindergarten teachers for parents are. Teachers need to make sure they are aware of the specific challenges that children face when they start school, as well as how they can help them adapt to this transition. It is their role to support families in coping with stress and expectations during the transition process through communication, information and active parenting. Moreover, many teachers agree that the educational level and the socio-economic level of parents influence the successful outcome of the transition of children to primary school. The level of parenting is believed to have a great impact on educational outcomes and is a determining factor in the child’s future development and well-being. Furthermore, many studies have shown that there is a remarkable relationship between family’s income and socio-economic situation, and the achievement of school literacy and thus the smooth transition of children to Primary school [37,40,41,42]. 

The overwhelming majority of sample teachers agree that parental support for the new situation experienced by their child facilitates childern’s transition from kindergarten to primary school. When parents often talk to their children, they encourage them, strengthen them, help them, and pay close attention to how they use their time, and they provide a warm and encouraging support framework [43].

Almost half of the sample teachers agree that parents should offer learning experiences at home, by reading books and playing educational games. Similarly, they agree that active involvement of parents in the school curriculum is essential in a transition program. In order for parents to be more involved in school [37], parents are advised to be better informed about the specific challenges faced by children who start school. Therefore, building a positive collaborative climate between kindergarten teachers, elementary school teachers and the family can help achieve the goal [5,44,45].

Parents know their children well and can make a valuable contribution to a transition program. This could be done by inviting parents to participate in the program [42,46] to include their opinions on the design and operation of the program [47] and to share ideas and information about their children’s transition. 

Approximately two out of five teachers agree that parents need to have time every day to help their children with school activities at home and supervising their school tasks [48,49] enhancing this way their school performance [19]. It is preferable parents to know what children learn at school and how they can continue their children’s learning at home. Unfortunately, more and more parents no longer have the time to actively participate in the education of their children [50]. 

The results of the survey showed that teachers believe that there are many good practices related to the smooth transition of children from Kindergarten to Primary School. The most common practice for all three groups of teachers is that parents should be invited to primary school for briefing at the beginning of the school year. In almost all schools in Greece, this meeting takes place with all parents, and international research findings seem to be applied with the same frequency in other countries [12,51]. 

Then we see that teachers consider a good and useful idea parents and children to visit Primary School before the beginning of the school year [22,51,52] to familiarize themselves with the new school environment and teachers, and to discuss their expectations, hopes and questions about education [2,49]. This way, the basis for an honest and regular communication is created. Undoubtedly, it is very important to provide parents with information about school very early. Information should also focus on the general behavior of children. In particular, they should seek to identify specific interests and/or problems and, above all, to create the conditions for decision-making in relation to the joint action of family and school. In this way, it is expected that the particularities of each child will be approached effectively.

Teachers believe that joint meetings of kindergarten teachers and primary school teachers with parents are extremely useful before the beginning of the school year. Mutual support between the parents in both Kindergarten and Primary School is also pointed out as a good practice of transition in this survey since parents expressing their own concerns, problems and expectations has much to offer [42,46,51]. 

Two very common practices applied internationally are sending brochures or letters describing what parents could do to prepare their children for school as well as telephone communication with the family [12,22,49,52]. These practices do not seem to be shared by Greek teachers who prefer live meetings and communication with their pupils’ parents.

Teachers’ less approved practice is visiting pupils’ homes at the beginning of the school year to get to know the child and the family. The results show serious hesitations on the part of the participants to take this action, although a visit to children’s home would allow teachers to collect useful information about children’s background and culture in order to support them individually in their transition, which contradicts with previous research efforts [5,49,53,54,55,56,57]. It seems that Greek teachers do not particularly agree with visits to children’s homes, perhaps because they feel that this is not their responsibility. It would be very interesting to explain the reasons why they do not agree with this practice in another study.

In conclusion, the assistance and support children receive from their parents and teachers for their transition to elementary school enhances their mental tolerance. Working closely with them and boosting their self-esteem and the ability to integrate new experiences into their belief system help children eliminate stress and gain attitudes and values throughout their lives.

It is a common assumption that transitions are successful when they rely on relationships between all the interested parties. The regular and mutual communication of all the interested parties in a climate of trust and mutual appreciation will facilitate positive social interactions and relationships and will lead to positive results, helping the smooth adaptation of the child to the new environment. It is also necessary for modern society, with its diverse social, cultural, and linguistic features, to adopt systemic models of cooperation and promote flexible approaches of transition.

Many aspects of a child’s social environment, including socio-economic, cultural, and family factors, have a major impact on educational outcomes and are key factors in a child’s future development and well-being. Getting to know the family, recognizing the needs of the children, and supporting them with appropriate strategies are therefore essential.

In the context of our present research, we found that teachers in Greece recognize the importance of parents’ participation in the educational transition of their children to elementary school. We have outlined their views on the priorities to be given to the involvement of parents in the transitional process. The use of a closed-ended questionnaire limits the nature and extent of the information provided by the respondents. A complementary qualitative approach (interview, case study), based on the findings of the present national research, will help to investigate and understand the answers.

## Figures and Tables

**Table 1:** Absolute (f) and relative (%) frequencies of teachers’ answers who agree on the prerequisites for a successful transition of children from kindergarten to primary school.

Prerequisites for a Successful Transition of Children from Kindergarten to Primary School	“YES”	
	F	%
Mutual trust and respect among all the interested parties (teachers, children, and parents).	1185	74.0
Parents’ participation in the transition process.	669	41.8
Children’s and families’ expectations on school should be taken into account.	481	30.0

**Table 2:** Absolute (f) and relative (%) frequencies of teachers’ answers who agree that the factors mentioned affect the successful outcome of the transition of children from kindergarten to the Primary School.

Factors that Affect the Successful Outcome of the Transition of Children from Kindergarten to the Primary School	“YES”	
	F	%
The level of trust of parents toward teachers	920	57.5
Parents’ opinion on school and learning	794	49.6
The educational level of parents	677	42.3
The socio-economic level of the family	519	32.4
Culture and language (ethnic minorities)	502	31.3

**Table 3:** Absolute (f) and relative (%) frequencies of teachers’ answers who agree that the factors that are involved in the preparation of children influence the successful outcome of their transition from kindergarten to the primary school.

Children’s Preparation for Primary School	«YES»	
	F	%
Parents’ support in the new situation that their children experience facilitates their transition from kindergarten to primary school.	1315	82.1
Parents must read books to their children and play math games at home regularly.	865	54.0
Parents’ active participation in the school curriculum is essential in a transition program from kindergarten to primary school.	772	48.2
Parents should dedicate time every day to help their children in school activities at home.	643	40.1
Parents should learn how to help their children learn to read.	601	37.5

**Table 4:** Absolute (f) and relative (%) frequencies, mean (M) and standard deviations (SD) of teachers’ opinions on the appropriate practices for the successful transition of children from Kindergarten to Primary School.

	«I Totally Disagree»	«I Disagree»	«Neutral Attitude»	«I Agree»	«I Totally Agree»	M	SD
	f (%)	f (%)	f (%)	f (%)	f (%)		
Parents are invited to Primary School for briefing at the beginning of the year	17 (1.1)	30 (1.9)	98 (6.1)	676 (42.2)	780 (48.7)	4.36	0.77
Parents and children visit Primary School before the beginning of the school year	90 (5.6)	150 (9.4)	382 (23.8)	678 (42.3)	302 (18.9)	3.59	1.07
Joint meetings of kindergarten teachers and teachers with parents before starting Primary school	107 (6.7)	261 (16.3)	501 (31.3)	536 (33.5)	197 (12.3)	3.28	1.09
Meetings and communication between parents of kindergarten and Primary School children	158 (6.6)	241 (15.0)	599 (37.4)	453 (28.3)	204 (12.7)	3.26	1.07
Brochures or letters to the family	114 (7.1)	273 (17.0)	531 (33.1)	566 (35.3)	118 (7.4)	3.19	1.03
Telephone communication with the family	143 (8.9)	241 (15.1)	516 (32.3)	573 (35.8)	127 (7.9)	3.19	1.07
